# Population-based surveys and monitoring of noncommunicable diseases

**DOI:** 10.1590/S1518-8787.201705100supl1ap

**Published:** 2017-06-01

**Authors:** Deborah Carvalho Malta, Célia Landmann Szwarcwald

**Affiliations:** I Universidade Federal de Minas Gerais. Belo Horizonte, MG, Brasil; II Instituto de Comunicação e Informação Científica e Tecnológica em Saúde. Fundação Oswaldo Cruz. Rio de Janeiro, RJ, Brasil

National population surveys are essential to learn the health profile, the distribution of risk factors and their tendencies, as well as health inequalities. The information collected periodically allow the monitoring of actions and programs and of health in different population subgroups and subsidize the planning and management of collective health interventions ^1 - 3^ . Recently, Brazil has stood out by the improvement and advances in the health information systems, as well as in the organization of epidemiological surveys ^1 - 3^ .

This supplement of *Revista de Saúde Pública* has articles based on unpublished findings of two major national health surveys: National Survey Health (PNS) ^4^ and Evaluation of Risk and Protective Factors for Chronic Diseases by Telephone Survey (VIGITEL) ^5^ .

National Health Survey was held in 2013, being performed by the IBGE in partnership with the Brazilian Ministry of Health and collaboration of various institutions of education and research. More than 1,000 technicians of IBGE participated in the collection of data on 1,600 Brazilian municipalities. The PNS is a home-based and cross-sectional study, with stratified sampling in three stages of conglomeration. The census tracts were the primary sampling units, households, second stage units, and adult residents (18 years or older), third stage units. We collected information from 64,348 households. In each household, an adult resident aged 18 years or older was selected by simple random sample and, in total, 60,202 individuals were interviewed ^3 , 4^ . PNS is the most extensive health survey ever held in the Country and includes themes such as health inequalities, use of services, primary health care, social assistance, health insurance, oral health, lifestyles, noncommunicable diseases, violence, accidents, life cycles (adults, older adults, children, women), people with disabilities and the use of medicines, including Anthropometry and blood pressure measures. In subsample, biochemical exams were collected ^3 , 4 , 6^ .

Of the 11 studies held with the data of PNS, Stopa et al. ^7^ highlighted the social inequalities in the use of health services. The authors ^7^ found that, of the individuals who sought the health service in both weeks, 95.3% used the services, although it was more used by people aged 60 years or older, who reside in the south and southeast regions and by those whose head of household had a higher level of education. Malta et al. ^8^ examined the factors associated with the use of services in the adult Brazilian population with non-communicable diseases (NCD), and Azevedo and Silva et al. ^9^ analyzed the actions of early detection for breast cancer, initiated with the medical request for mammography. These authors ^8 , 9^ identified differences between users of the Brazilian Unified Health System (SUS) and those who have private health insurance. However, Melo Silva et al. ^10^ showed that the functional impairment in the older adults had a strong association with the number of medical appointments and hospitalizations, being similar in both public and private health systems.

Still among the articles that addressed the NCD, Malta et al. ^11^ analyzed the factors associated with chronic pain in the column, concluding that advanced age, excess weight and obesity are important predictors for both sexes. Another article ^12^ analyzed factors associated with self-reported diagnosis of diabetes and found association with age, low education, smoke in the past, overweight and obesity, and high blood pressure, as well as with health state reported as bad. Barros et al. ^13^ identified that individuals with depression have more unhealthy behaviors. Ahmed et al. ^14^ , on the other hand, in the article on work-related musculoskeletal disorder (DORT/LER), stressed the importance of this occupational disease that is the main cause of absences from work and results in disability in adults.

Regarding the analysis of inequalities in the health state of the Brazilian population, the article by Szwarcwald et al. ^15^ showed relevant geographical differences in healthy life expectancy, indicator that measures the expected number of healthy life years for the population of a given age. As for the older adults, Lima-Costa et al. ^16^ estimated the prevalence of informal and paid help to individuals with 60 years or older with functional limitations, which presented association with sociodemographic factors.

Jaime et al. ^17^ , on the other hand, identified the family influence and living conditions in the consumption of sugary drinks in children under two years of age.

The VIGITEL survey has been deployed since 2006 in the 26 Brazilian capitals and the Federal District, completing 11 years of continuous collection. The coordination is from the Health Surveillance Secretariat, MS, in partnership with the Center for Epidemiological Research in Nutrition and Health (NUPENS) of the University of São Paulo and support from several teaching and research institutions in the Country. With computer-aided telephone interviews, VIGITEL uses probabilistic samples of the adult population aged 18 years or older, residing in households served by at least one fixed telephone line in the year of the survey, in each one of the 26 capitals of Brazilian states and in the Federal District, totaling more than 54,000 interviews annually. Post-stratification weights are used to correct estimates ^5^ . Of both articles presented in this supplement based on analysis of information collected on VIGITEL 2015, the study by Bernal et al. ^18^ aimed to assess the impact on the changes of the prevalence of chronic diseases’ risk factors, disclosed on Vigitel, after the inclusion of data from the population that does not have fixed telephone line, only mobile phone. The other article analyzed the factors associated with the self-reported diagnosis of arterial hypertension ^19^ .

The results presented here extend the knowledge of the epidemiological profile of the Brazilian population, providing grants to states and municipalities for the planning of health promotion actions, being characterized as a health surveillance tool. Many of the articles have addressed issues regarding NCD, which represents a health problem of great magnitude, leading to premature deaths, disabilities, and negative impacts on the economy of the Country ^20^ . The WHO recommends the organization of NCD surveillance by the monitoring of diseases and their risk factors, aiming to support actions for prevention and control ^20 , 21^ .

The importance of these surveys’ sustainability is important to highlight, as well as the compromise with the continuity of these research, which will play the role of monitoring of public policies and, in particular, of indicators and targets for reduction and control of NCD ^20 , 22 , 23^ .
